# Chemical Characterization of Taif Rose (*Rosa damascena*) Methanolic Extract and Its Physiological Effect on Liver Functions, Blood Indices, Antioxidant Capacity, and Heart Vitality against Cadmium Chloride Toxicity

**DOI:** 10.3390/antiox11071229

**Published:** 2022-06-23

**Authors:** Reham Z. Hamza, Njood A. Al-Malki, Sarah Alharthi, Saif A. Alharthy, Bander Albogami, Samy M. El-Megharbel

**Affiliations:** 1 Biology Department, College of Sciences, Taif University, P.O. Box 11099, Taif 21944, Saudi Arabia; s43713629@students.tu.edu.sa (N.A.A.-M.); b.boqami@tu.edu.sa (B.A.); 2 High Altitude Research Canter, Taif University, P.O. Box 11099, Taif 21944, Saudi Arabia; 3 Chemistry Department, College of Sciences, Taif University, P.O. Box 11099, Taif 21944, Saudi Arabia; sarah.alharthi@tu.edu.sa (S.A.); s.megherbel@tu.edu.sa (S.M.E.-M.); 4 Department of Medical Laboratory Sciences, Faculty of Applied Medical Sciences, King Abdulaziz University, P.O. Box 80216, Jeddah 21589, Saudi Arabia; saalharthy@kau.edu.sa; 5 King Fahd Medical Research Center, King Abdulaziz University, P.O. Box 80216, Jeddah 21589, Saudi Arabia

**Keywords:** Taif Rose, cadmium chloride, hepatotoxicity, injured cardiac tissues, oxidative stress

## Abstract

Exposure to cadmium chloride (CdCl_2_) causes an imbalance in the oxidant status of the body by triggering the release of reactive oxygen species (ROS). This study investigated the effect of *Rosa damascena* (*R. damascena*) extract on oxidative stress, hepatotoxicity, and the injured cardiac tissue of male rats exposed to CdCl_2_. Forty male Wistar albino rats were divided into four groups: the vehicle control (1 mg/kg normal saline), the CdCl_2_-treated group (5 mg/kg), the *R. damascena* extract group (100 mg Kg), and the combination of CdCl_2_ and *R. damascena* extract group. Male rats exposed to CdCl_2_ showed multiple significant histopathological changes in the liver and heart, including inflammatory cell infiltration and degenerative alterations. Successive exposure to CdCl_2_ elevated the levels of hepatic and cardiac reactive oxygen species (ROS), malondialdehyde (MDA), tumour necrosis factor-alpha) (TNF-α) and interleukin -6 (IL-6) and decreased antioxidant defences. The extracts significantly increased the levels of glutathione, superoxide dismutase (SOD), and catalase (CAT), whereas it dramatically decreased the levels of lipid peroxidation (LPO), alanine aminotransferase (ALT), aspartate aminotransferase (AST), and the mRNA of TNF-α and IL-6. *R. damascena* administration prevented liver and heart injury; suppressed excessive ROS generation, LPO, and inflammatory responses; and enhanced antioxidant defences. In addition, *R. damascena* upregulated the mRNA of TNF-α and IL-6 in CdCl_2_-administered male rats. In conclusion, *R. damascena* modulated the oxidative stress and inflammation induced by CdCl_2_. The hepatic and cardiac tissue damage and histopathological alterations resulting from the CdCl_2_-induced oxidative stress were counteracted by the administration of *R. damascena* extracts. *R. damascena* enhanced antioxidant defence enzymes in male rats.

## 1. Introduction

Cadmium (Cd) is a heavy metal and is non-essential in mammals. It can cause severe and toxic effects on soft organs and may affect many physiological functions [1]. Cadmium chloride (CdCl_2_) is a toxic substance that threatens human health, according to the International Agency for Research on Cancer, which has classified CdCl_2_ as a Group I carcinogen [2]. The problem caused by CdCl_2_ has been increasing and is adversely affecting human health [3,4]. Free electrons, known as free radicals, are the main source of severe oxidative stress induced by various CdCl_2_ sources, such as cigarette smoking, contaminated food and drinking water, and industrial applications such as CdCl_2_ batteries [5].

Heavy metal pollution is a significant environmental problem. The use of industrial products that contain cadmium metal is increasing, leading to environmental pollution and increased occupational exposure and toxicity in humans. Exposure to CdCl_2_ can result in negative health effects, such as cardiovascular diseases, alterations in organ function, and hepatotoxicity [6].

Aside from occupational exposure to CdCl_2_, tobacco consumption accounts for approximately 90% of CdCl_2_ exposure. The rate of absorption of CdCl_2_ intake through diets is estimated to be 3–5% in humans; however, some studies have suggested that it may be as high as 44% [6].

CdCl_2_ is a cumulative toxicant, with a long half-life that can reach 10–33 years in humans. This can result in a biological accumulation of CdCl_2_ in multiple organs and an elevated burden on the body over time [7]. After absorption, CdCl_2_ is distributed throughout the body primarily through blood circulation [8,9]. CdCl_2_ alters the binding positions of metal ions, such as Ca^2+^, Zn^2+^, and Fe^3+^, on the membrane transport proteins, altering the permeability of cellular membranes and increasing its entry into the cells [10]. This duplicates its toxic effect on the liver and induces cardiac damage, cardiovascular disease, and immune system disorders [11].

Oxidative stress plays an important role in facilitating the toxicity of CdCl_2_ [12,13]. Cadmium exposure results in increased levels of the final markers of lipid peroxidation (LPO), a reduced cellular defence mechanism in rats, a redox imbalance in blood and organ tissues, and oxidative DNA damage [14,15,16,17]. Therefore, alleviating oxidative injury may reduce the deleterious effects of CdCl_2_ exposure.

Many studies have demonstrated that free radicals play vital roles in maintaining human health. An imbalance in the scavenging activity of reactive oxygen species (ROS) may result in severe oxidative stress, leading to serious and excessive oxidative injury to biomolecules, such as DNA, lipids, and proteins. Many chronic diseases have been associated with the existence of continuous severe oxidative stress [18,19]. Considering different formulations, the herbal varieties have the potential for free radical scavenging and have gained considerable attention for the treatment of disease. Therefore, the interest in finding natural products with antioxidant activities without side effects has increased significantly.

Phytochemicals with antioxidant activities due to the presence of phenolic compounds are receiving increased attention due to their efficacy in the prevention of many diseases. The genus *Rosa* is considered the queen of flowers. *Rosa* includes many species that are distributed worldwide. The *rose* is one of the largest and most important crops, is used as a garden plant, and is occasionally used as cut flowers. *Rose* products have also been used in the perfumery and food sectors for many years [20].

*R. damascena* is a species of high importance [21]. Taif roses have been processed into rose water for the past two centuries. Taif rose oil and rose water are very important and commercially valuable products [21]. The main products of rose are dried petals, rose water, and rose oil, all of which are used in the food, cosmetics, and perfume industries [22,23]. Rose species have been reported to have anti-human immunodeficiency virus, as well as anti-inflammatory and antidepressant properties [24].

The rose species Ward Taifi, also known as Taif rose (*R. damascena*), is considered one of the most important products of the Taif governorate in the kingdom of Saudi Arabia. In the current study, the biological activity of Taif rose extract was investigated. The total phenolics and flavonoids were estimated, and a histological investigation of the gene expression of some antioxidant enzymes was conducted.

The daily use of permanent and natural resources, such as plants, increases the discovery of new therapeutic agents. Medicinal plants are the richest source of traditional medicines, food supplements, and pharmaceutical intermediates for synthetic drugs [25,26].

Although the beneficial effect of the Taif rose extract has been reported previously, no previous study has reported its ability to alleviate hepatic and cardiac injuries in male rats exposed to CdCl_2_. Considering the importance of this genus of rose specifically for the Taif governorate, novel metabolites were determined to confirm the new concept of this study. Therefore, the current study was designed to assess the severe toxic effects of CdCl_2_ alone or in combination with the *R. damascena* extract. This study assessed the toxicity of CdCl_2_ on liver function markers and heart vitality, on enzymes such as superoxide dismutase (SOD) and catalase (CAT), and on a non-enzymatic antioxidant marker—namely, malondialdehyde (MDA). In order to clarify the protective effects of *R. damascena* against CdCl_2_ exposure, which causes oxidative injury and tissue damage in the hepatic and cardiac tissue of male rats.

## 2. Materials and Methods

### 2.1. Plant Sampling

*R. damascena* was identified morphologically and based on the production yield. *R. damascena* leaves were selected due to their potential medical benefits. Rose plants of various sizes were selected from the Taif rose farm in the Al-Shafa Highlands in Taif, Saudi Arabia, on 15 October 2020. Taif roses were collected and weighed. The biomass was determined by multiplying the average individual weight by the number of individuals per farm.

### 2.2. Preparation of R. damascena Water Extract and Determination of Flavonoids, Total Phenolics, and ROS Free Radical-Scavenging Activity

*R. damascena* leaves were finely ground to powder, 100 g of which was soaked in 1 L of 80% cold methanol for approximately 48 h and dried using a rotary evaporator. This extract was divided into parts to perform each test. The contents of polyphenols and flavonoids in the prepared extract were assayed using the Folin–Ciocalteu method [27] and aluminium trichloride method [28], respectively. To evaluate the ROS-scavenging activity of the *R. damascena* extract, we conducted a 2,2-diphenyl-1-picryl-hydrazyl (DPPH) radical assay [29] using DPPH purchased from Sigma, St. Louis, MO (USA).

### 2.3. Extraction of R. damascena and Determination of Its Active Components

*R. damascena* leaf samples were extracted with methanol (MeOH) (high-performance liquid chromatography (HPLC) grade) by ultrasonication for 30 min, three times. The mixture was then filtered, and the extract was collected, evaporated under vacuum at 50 °C, and stored in a deep freezer.

### 2.4. HPLC Analysis

A Waters 2690 Alliance HPLC system was equipped with a Waters 996 photodiode array detector. The extract (rose MeOH extract, 93 mg/mL) was dissolved in methanol and filtered using a 0.22 μm syringe filter, and 10 μL was injected. Stock solutions of 10 different standards in methanol were prepared. Each of the standards was filtered using a 0.22 μm syringe filter, and then, 10 μL was injected. HPLC analysis conditions were as follows: Column C18, Inertsil ODS 3: 4.6 × 250 mm, 5 μm; mobile phase (A): buffer (0.1% phosphoric acid in water) and methanol (65% water: 35% methanol); mobile phase (B): buffer (0.1% phosphoric acid in water) and methanol (50% water: 50% methanol); mode of elution: gradient; flow rate: 1 mL/min; temperature: ambient; and wavelength: 280 nm. The HPLC conditions and optimisation timetable are shown in Table 1.

### 2.5. Experimental Animals

Two-month-old male Wistar albino rats were included in this study and were housed under standard temperature conditions and supplied with food ad libitum.

**Figure 1:**
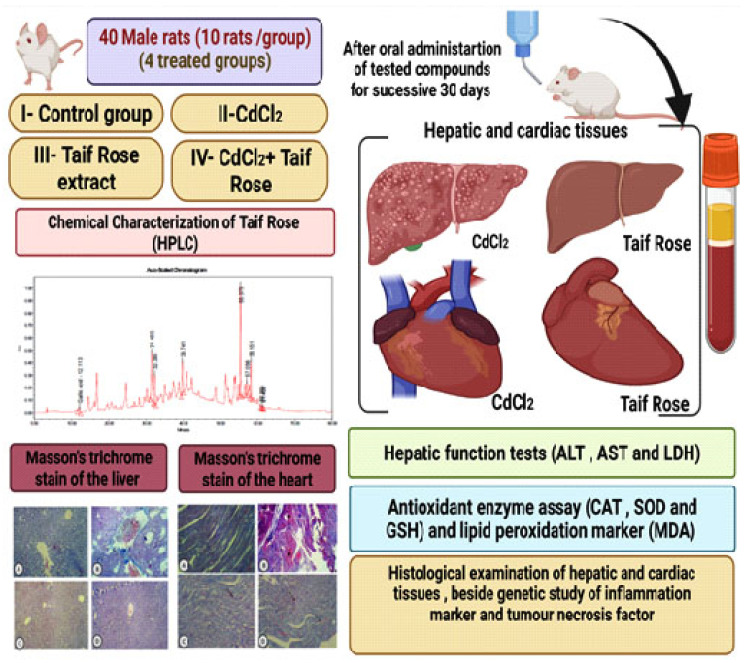
Experimental protocol.

The rats were divided into four treatment groups, with 10 in each group, and numbered for the oral administration of samples. The weight of the same rat was recorded in the experiment as follows (Figure 1): Group 1 (control group) received 1 mL/kg of the saline solution orally. Group 2 received the *R. damascena* extract (100 mg Kg^−1^) orally. A stock solution was prepared from the extract (24 µg/100 mL) (residual weight). The rats were weighed weekly and the required dose (100 mg Kg^−1^) of the prepared stock solution was calculated for each animal. The determination of a safe dose that does not induce mortality was based on a previous study by Hamza et al. [30]. The dose required for each rat was determined in a weight–dose-dependent manner, and the weights of the animals were recorded weekly to adjust the oral dose upon weighing [30] for 30 consecutive days (*R. damascena*). Group 3 received 5 mg/kg of CdCl_2_ via oral gavage [7], dissolved in the saline solution (CdCl_2_), and Group 4 received the same doses of CdCl_2_, followed by *R. damascena* after 30 min of oral administration (*R. damascena* + CdCl_2_) at the same doses via an oral tube.

Blood samples were collected and centrifuged at 10,000× *g* rpm for approximately 20 min to obtain serum samples for the hepatic enzymes assay alanine aminotransferase (ALT) and aspartate aminotransferase (AST). The male rats were immediately dissected, and the hepatic and cardiac tissues were collected. The tissue samples were fixed in approximately 6% of neutral buffered formalin for the examination of the histopathological sections. Other hepatic samples were kept at −80 °C for mRNA isolation. Furthermore, other samples were successively homogenised in phosphate-buffered saline (each gram of tissue immersed in 5 mL of PBS) at 4 °C, and the supernatant was stored at −80 °C.

### 2.6. Biochemical Assays

The ALT and AST enzymes in the serum were evaluated by using commercial kits (Spinreact, Barcelona, Spain). MDA, a final lipid peroxidation marker, was assayed in the hepatic and cardiac tissues [31], and absorbance was measured spectrophotometrically at 532 nm. GSH was performed as the reaction was monitored indirectly as an oxidation rate of NADPH at 240 nm for 3 min and expressed as nmol/100 mg protein [31]. SOD was measured by assaying the autooxidation of pyrogallol at 440 nm for 3 min and expressed as U/g [32]. CAT activity was assessed by assaying the hydrolysis of H_2_O_2_ and the resulting decrease in absorbance at 240 nm over a 3 min period. The activity was expressed as U/g [33,34]. All antioxidant markers were evaluated in the homogenates of the liver and heart tissues of all treated groups.

### 2.7. Determination of Inflammation Markers

Tumour necrosis factor-alpha (TNF-α) and interleukin 6 (IL-6) were assayed in the serum of male rats using ELISA kits (Cat. Nos R6365 and RB1829), BIOTANG INC., Lexington, MA, USA) in accordance with the manufacturer’s instructions. IL-6 was quantified using an anti-canine monoclonal antibody (mAb) and biotinylated polyclonal antibody. The TNF-α test was developed, and plates were read with a spectra count apparatus using a 450 nm filter. The minimum sensitivity for detecting IL-6 was 78 pg/50 μL, and that for TNF-*α* was 3.12 pg/50 μL.

### 2.8. Histopathological Study

The liver and heart samples were fixed in 10% neutral buffered formalin for 48 h and were processed for examination by haematoxylin and eosin (H&E) staining [35]. The tissues were examined under a light microscope.

### 2.9. Masson’s Trichrome Stain Study

Masson’s trichrome is a three-colour staining protocol for distinguishing cells from surrounding connective tissue. The stain produces red keratin and muscle fibres, blue or green collagen and bone, light-red or pink cytoplasm, and dark brown-to-black cell nuclei. The trichrome was applied through immersion of the fixated sample into Wiegert’s iron haematoxylin [36].

### 2.10. Gene Expression

The effect of *R. damascena* on the mRNA of TNF-α and IL-6 was determined via qRT-PCR. Total mRNA was isolated from the hepatic tissue samples of the male rats using TRIzol reagent. The isolated mRNA was quantified and selected for cDNA synthesis. SYBR green master mix (Fermentas, Fairview Ave, Roseville, CA, USA), and the primers listed in Table 2 (Vivantis Technologies, Shah Alam, Selangor, Malaysia) were used for cDNA amplification with Rt-PCR. The obtained data were analysed using the 2^-ΔΔCt^ method [37].

### 2.11. Statistical Analysis

The results are presented as mean ± SE using Statistical Package for Statistical Sciences (SPSS) software version 27 IBM Corp: Armonk, NY, USA, 2020 [38] and Open Epi version 2.3.1. One-way ANOVA and post hoc power were used to analyse the data. A value of *p* < 0.05 was considered significant (using three repetitions).

## 3. Results

### 3.1. Total Phenolics, Free Radical Scavenging, and Flavonoids of R. damascena

**Figure 2:**
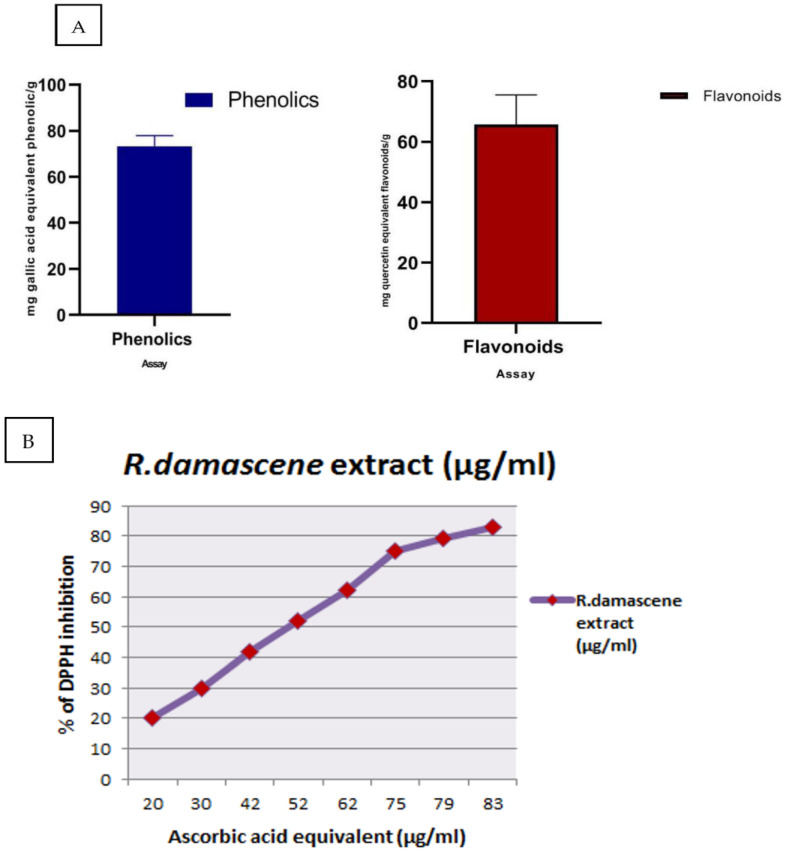
(**A**) Total phenolics and flavonoid content; (**B**) scavenging activity of *R. damascena* against DPPH. Data are expressed as mean ± SE.

The evaluation of total phenolics and total flavonoids (Figure 2A) identified that the extract of *R. damascene* contained 73.32 ± 1.87 mg of gallic acid equivalent phenolic/g and 65.70 ± 1.94 mg of quercetin equivalent flavonoids/g. The free radical scavenging activity against DPPH contributed to the efficacy of *R. damascena* (Figure 2B).

### 3.2. HPLC of R. damascena (MeOH)

**Figure 3:**
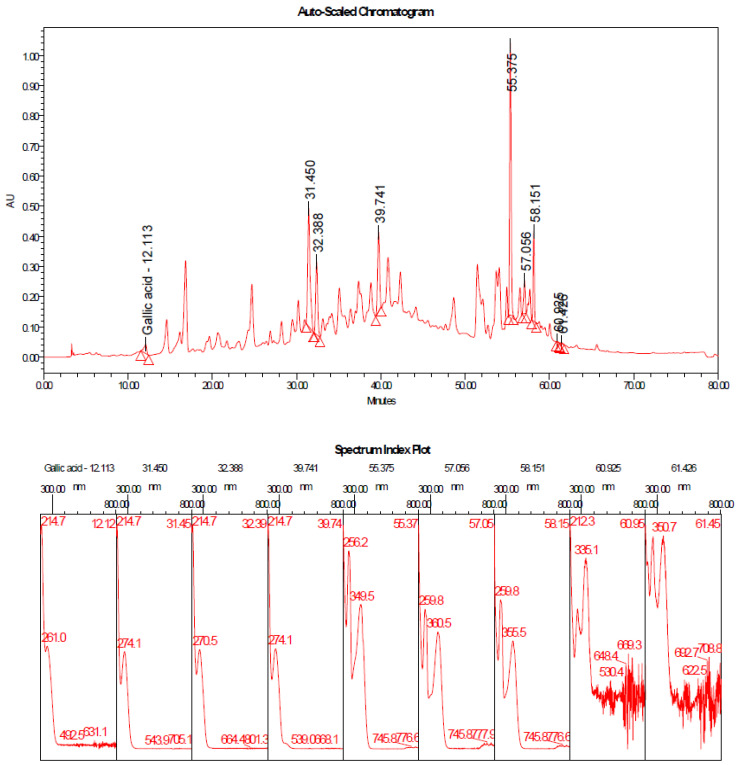
HPLC analysis and spectrum index plot of Taif Rose (*R. damascena*) methanolic extract at 280 nm.

The results of the HPLC analysis (Figure 3) of the Taif rose (MeOH) extract showed the presence of various active constituents, such as gallic acid, apigenin, quercetin, and eugenol. The evidence acquired using chromatography at various retention times (Table 3) revealed the main constituents found in Taif rose (MeOH) extract.

### 3.3. R. damascena Alleviated Liver Injury in Rats Exposed to CdCl_2_

Excessive exposure to CdCl_2_ for 30 consecutive days resulted in a significant increase in the serum activity of ALT and AST. In contrast, the supplementation of *R. damascena* extracts after CdCl_2_ exposure resulted in a significant decrease in ALT and AST hepatic enzymes and alleviated hepatic toxicity induced by CdCl_2_ (Table 4).

### 3.4. R. damascena Alleviated Oxidative Injury in the Liver and Heart of Male Rats Exposed to CdCl_2_

There was a marked elevation (*p* < 0.001) in MDA levels in the hepatic (Table 5) and cardiac (Table 6) tissues of the CdCl_2_-treated group. The supplementation of the *R. damascena* extracts significantly decreased (*p* < 0.05) the hepatic and cardiac MDA levels in the CdCl_2_-treated groups.

In contrast, in the groups exposed to CdCl_2_, hepatic (Table 5) and cardiac (Table 6) tissues exhibited significant decreases (*p* < 0.05) in GSH content, SOD activity, and CAT activity, compared with those of the control group. Oral supplementation of *R. damascena* extracts elevated the levels of antioxidant enzymes (SOD and CAT) in hepatic (Table 5) and cardiac (Table 6) tissues of CdCl_2_-treated rats.

### 3.5. R. damascene (100 mg Kg^−1^) Alleviated IL-6 and TNF-α in Groups Exposed to CdCl_2_

The levels of TNF-α and interleukin- 6 (IL-6) were significantly (*p* ˂ 0.001) higher in the serum of the CdCl_2_ group than those observed in the normal control group. *R. damascena* administration significantly reduced all the concentrations of these molecules. Therefore, *R. damascena* was highly efficient and could restore the concentrations of inflammatory markers to normal levels, similar to those in the control group (Table 7).

### 3.6. R. damascena Mitigated Inflammatory Marker Expressions

**Figure 4:**
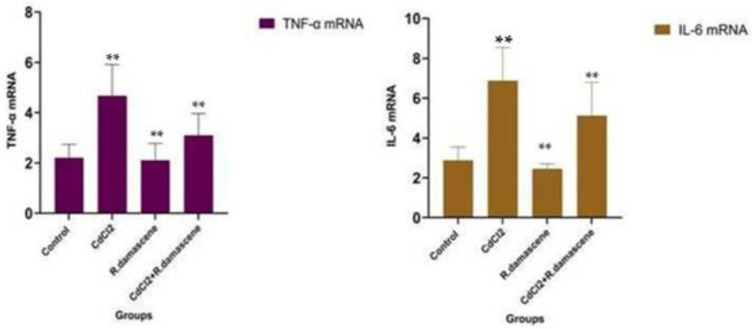
*R. damascena* leaves extract dose (100 mg Kg^−1^) increased the expression of TNF-α and IL-6 in the hepatic tissues of CdCl_2_ (5 mg Kg)-exposed male rats. Data are expressed as mean ± SE. ** means that the difference was highly significant (*p* < 0.01).

The results revealed that changes in hepatic TNF-α and IL-6 expressions mainly determined the effects of *R. damascene* on CdCl_2_-induced severe inflammatory response. The findings also unveiled the marked upregulation of TNF-α and IL-6 mRNA in hepatic tissues of CdCl_2_-treated group, as shown in Figure 4.

### 3.7. Histological Examination

**Figure 5:**
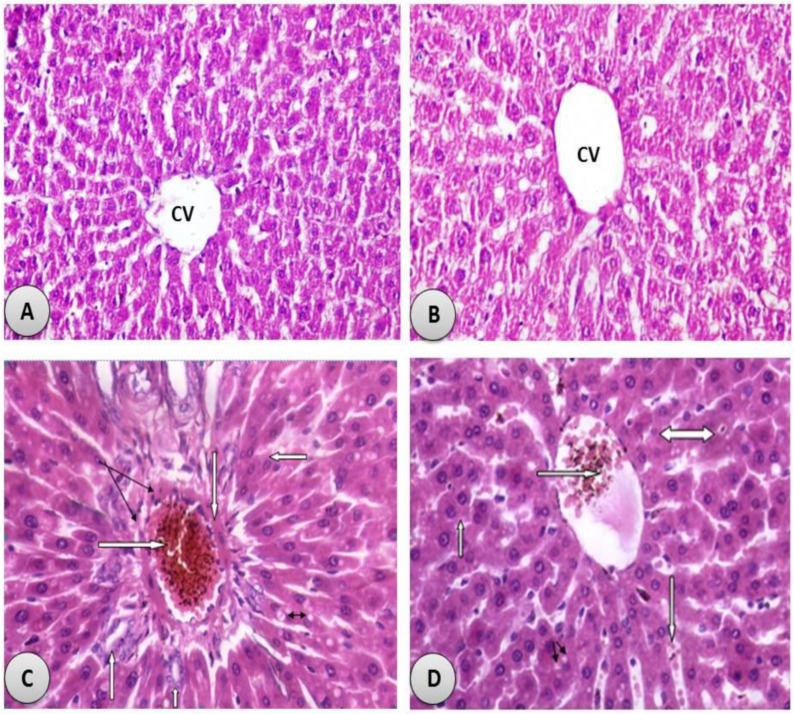
Photomicrograph of H&E-stained sections in the liver: (**A**) control group showed normal hepatic cells and the normal central vein (thin arrow) (H&E × 400); (**B**) *R. damascene*-treated group showed normal hepatic structures with normally sized central vein (CV) (H&E × 400); (**C**) CdCl_2_ treated group showing toxicity in the form of hypertrophy of hepatocytes with appearance of binucleated hepatocytes and increased eosinophilia, granular cytoplasm and vesicular nuclei (Right forwarded white arrow), the portal tract shows severe congestion of the portal vein which is filled with haemorrhage and necrotic tissues ( White forwarded white arrow), ductular reaction (new bile ducts formation) at the periphery of portal tract (Black arrow) with perivenular fibrosis and mild hyalinisation in its wall (Upward white arrow), infiltration by inflammatory cells of the portal tract (Two headed arrow) and ballooning degeneration in some hepatocytes (H&E × 400). (**D**): CdCl_2_ combined with *R. damascena* showing hepatic tissues with mild toxicity in the form of hypertrophy of hepatocytes with granular eosinophilic cytoplasm and vesicular nuclei and appearance of some binucleated cells, with mild congested central vein, ballooning degeneration of some hepatocytes (Two sided arrow) and few dilated congested blood sinusoids (H&E × 400).

The histological examination confirmed the hepatoprotective effects of the administration of *R. damascena*. The control (Figure 5A) and *R. damascena*-supplemented (Figure 5B) groups showed normal hepatic and hepatocyte structures. The CdCl_2_-exposed male rats showed infiltration of the inflammatory cells, congestion of the central veins, and degenerative structural changes (Figure 5C). The administration of *R. damascena* markedly inhibited CdCl_2_-induced hepatic histological alterations (Figure 5D). 

**Figure 6:**
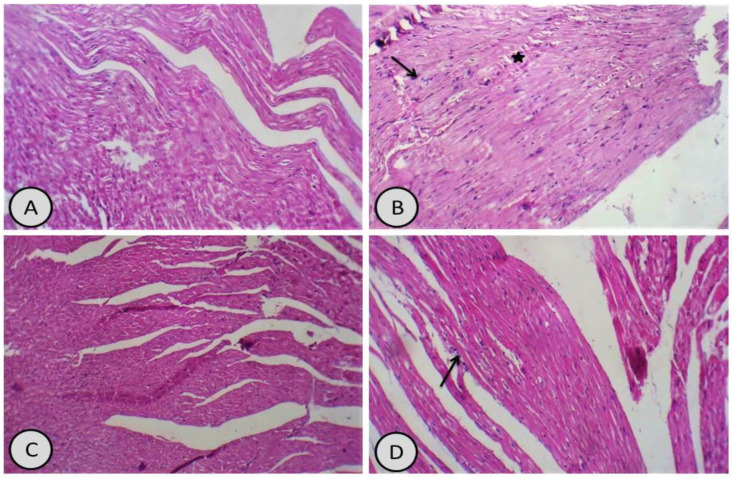
Photomicrographs of heart sections: (**A**) control group showed normal cardiac muscles and fibres (H&E × 400); (**B**) CdCl_2_ group showed interstitial inflammatory cells (arrow) and muscle fibre disruption with interstitial haemorrhage (*) (H&E × 400); (**C**) *R. damascena* group showed normal cardiac muscle fibres (H&E × 400); (**D**) group treated with *R. damascena* in combination with CdCl_2_ showed restoration of cardiac muscle structure with appearance of interstitial inflammation (thin arrow) H&E × 400).

For the cardiac tissues, the control group showed normal cardiac muscles and fibres (Figure 6A). Meanwhile, CdCl_2_-treated group showed interstitial inflammatory cells and cardiac muscle fibre disruption with haemorrhage (Figure 6B). *R. damascena*-treated group showed normal cardiac muscles (Figure 6C). The last treated group, treated with *R. damascena* in combination with CdCl_2_, showed restoration of cardiac muscles with some interstitial inflammation (Figure 6D).

**Figure 7:**
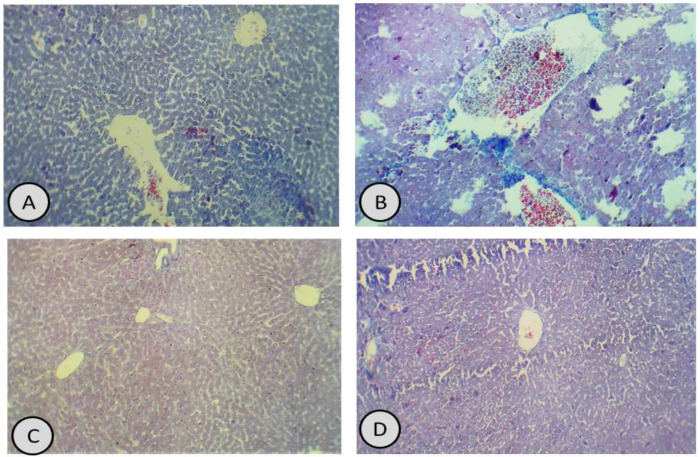
(**A**) Masson’s trichrome stain of the liver in control group showed normal amount of collagen fibres (×200); (**B**) Masson’s trichrome stain of the liver in CdCl_2_-treated group showed an increased amount of collagen deposition, compared with control rats (×200); (**C**,**D**) Masson’s trichrome stain of the liver in groups treated with *R. damascena* and combination of CdCl_2_ and *R. damascena* showed normal amounts of collagen fibres in the hepatic tissues (×200).

**Figure 8:**
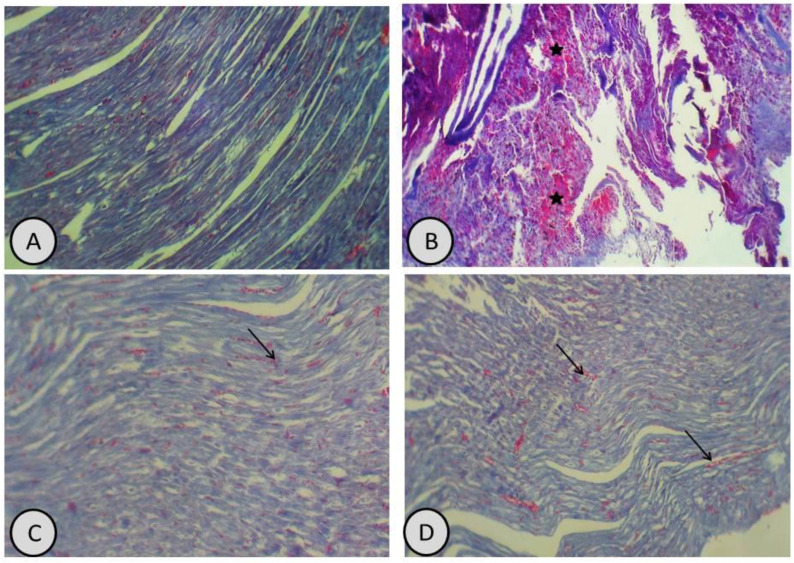
(**A**) Masson’s trichrome stain of the heart in control group showed normal amount of collagen fibres (×200); (**B**) Masson’s trichrome stain of the heart in CdCl_2_-treated group showed an increased amount of collagen deposition (Black stars), compared with control rats (×200); (**C**,**D**) Masson’s trichrome stain of the heart in groups treated with *R. damascena* and combination of CdCl_2_ and *R. damascena* showed normal amounts of collagen fibres with some mild deposition (Black arrow) (×200).

Furthermore, the sections with Masson’s trichrome staining showed lower deposition of collagen fibres in both hepatic and cardiac tissues, compared with increased deposition of collagen fibres in the hepatic tissues (Figure 7 and Figure 8).

## 4. Discussion

The results of the current study showed that Taif rose extract has a highly potent antioxidant effect on the amelioration of hepatic functions and heart vitality, as demonstrated by the high total phenolic content, biochemical analysis of liver enzymes, antioxidant enzyme markers, histological vitality, and the alleviation of hepatotoxicity induced by CdCl_2_. As reported previously, CdCl_2_ induces a marked reduction in protein levels and elevates the hepatic enzymes AST, ALT, and lactate dehydrogenase.

The current study revealed that Taif rose is rich in total phenolics and flavonoids. These findings are consistent with the results of Gala et al. [39], who demonstrated that the Taif rose contains high levels of flavonoid components, such as apigenin, luteolin, quercetin, rutin, and kaempferol, as well as phenolic components, such as ellagic acid, gallic acid, and phloroglucinol. 

The HPLC analysis of the Taif rose extract (*R. damascena*) revealed that the main constituents of this extract include nonadecane, phthalic acid, gallic acid, and quercetin. This finding is supported by the previous study of Hamza et al. [30], who demonstrated the presence of these compounds in addition to vitamin E and other constituents. Phenolic compounds have an important role as antioxidants. For example, gallic acid has been reported as a potent antioxidant against oxidative injury, such as reported by Gao et al. [40], who demonstrated that gallic acid is a part of plant metabolites that are widely spread throughout the plant kingdom. Gallic acid has strong antioxidant and free radical scavenging activities and can protect biological cells, tissues, and organs from damage caused by oxidative stress. These findings reinforce the results obtained in the current study.

HPLC also confirmed the presence of prasterone (dehydroepiandrosterone, (DHEA)). DHEA is a precursor hormone that is produced by the human body and is transformed into female and male sex hormones—namely, oestrogen and progesterone. Thus, it is an important constituent of *R. damascene* and is of great economic interest to Saudi Arabia.

Apigenin is found in the *R. damascena* extract and many other plants. It is a natural product belonging to the flavone class and is an aglycone of several naturally occurring glycosides [41]. Apigenin has been used as a traditional medicine for centuries because of its physiological functions as an antioxidant and anti-inflammatory, its role in lowering blood pressure, and its antibacterial and antiviral properties [42].

Another important constituent of the *R. damascena* extract is quercetin, which is a known, potent antioxidant and anti-inflammatory agent and greatly enhances immune capacity during pandemics such as the COVID-19 pandemic [43,44,45,46]. Eugenol, which is used as an ingredient in teas, perfumes, flavourings, and essential oils, was also detected [47]. It is also used as a local antiseptic and anaesthetic and can be used in dentistry applications.

Another study confirmed that the *R. damascena* extract can improve antioxidant capacity and reduce oxidative stress injuries in male rats receiving CdCl_2_. The *R. damascena* extract has high antioxidant content and can exert a protective effect against oxidative damage [48].

Hepatic dysfunction was observed in the CdCl_2_ group. This may be due to impaired protein synthesis, as reported by Chawla [49]. The decline in protein levels may have been due to liver dysfunction resulting from CdCl_2_ exposure and the alteration of the biosynthesis of proteins [39]. Gaskill et al. [40] reported severe hepatic injury, which was characterised by marked elevations in the levels of ALT, AST, and alkaline phosphate (ALP) hepatic enzymes. This was identified in the CdCl_2_-treated group; thus, these results are in accordance with the findings of the current study.

Our results are also in accordance with those of Newairy et al. [50], who reported that CdCl_2_ elevated the markers of oxidative stress. Oxidative stress is considered the first sign of severe hepatotoxicity.

Confirming the obtained results, Renugadevi and Prabu [51] reported that CdCl_2_ induced severe oxidative stress in the hepatic tissues of male rats. Liver damage caused by CdCl_2_ treatment was demonstrated by elevated levels of hepatic enzymes with elevated levels of LPO markers.

Lakshmi et al. [52] also reinforced these results by reporting that CdCl_2_ exposure increased the biomarker levels in the liver and decreased total protein levels. CdCl_2_ administration resulted in a significant increase in the TNF-α level. The present results are in accordance with those of Alghasham et al. [40], who reported that CdCl_2_ treatment significantly elevated the levels of TNF-α, IL-6, and oxidative stress markers in male rats. Therefore, CdCl_2_ toxicity induced a significant release of TNF-α and IL-6, which are associated with oxidative stress.

Our results revealed that Taif rose extract has characteristics that protect hepatic tissues and heart vitality and improve antioxidant status. Current results were previously confirmed by Hamza et al. [30], who reported that the main constituents of Taif rose methanolic extracts were vitamin E, octadecatrienoic acid, and hexadecanoic acid. All of these constituents are considered the main constituents responsible for the hepatoprotective effect and antioxidant activity of Taif rose extract.

Hamza et al. [30] demonstrated that methanolic waste extract of Taif rose improved liver histology and ultrastructure and inhibited the viability of hepatic cancer cells—hepatoma G2 cells (HepG2).

CdCl_2_ induces hepatic toxicity in addition to both genotoxicity and cellular injury in the hepatic tissues of treated animals. These findings are consistent with the results of the current study [52].

The *R. damascena* extract ameliorated CdCl_2_-induced hepatic and cardiac injury. Oral supplementation of the *R. damascena* leaf extract decreased the levels of serum aminotransferases and inhibited liver and heart injury, demonstrating its hepato- and cardioprotective effects. To our knowledge, this is the first report of both the hepatoprotective and cardioprotective effects of the *Taif R. damascena* extract against CdCl_2_ toxicity [53]. The Taif *R. damascena* extract ameliorated liver function markers, increased cardiac vitality, and prevented oxidative damage [54].

## 5. Conclusions

The current study presents new information on the cardio- and hepatoprotective effects of the *R. damascena* (Taif rose) leaf extract against CdCl_2_, which induced hepatic tissue damage in male rats. *R. damascena* prevented histopathological alterations in the hepatic and cardiac tissues and suppressed the LPO marker and inflammatory mediators in the hepatic and cardiac tissues resulting from exposure to CdCl_2_. Additionally, *R. damascena* decreased the hepatic and cardiac TNF-α and IL-6 levels and enhanced the potent antioxidant defence systems, thereby preventing CdCl_2_-elicited oxidative injury and severe inflammation. Therefore, *R. damascena,* which is endemic in the Taif region, is a candidate for attenuating hepatic and cardiac toxicity caused by CdCl_2_ exposure in male rats.

## Figures and Tables

**Table 1:** Timetable of HPLC conditions and optimisation.

Mobile Phase (A)	Concentration % of Eluent	Time (min)	Mobile Phase (B)	Concentration% of Eluent	Time (min)
Mobile phase (A) 65% water and 35% methanol	84%	0–10	Mobile phase (B) 50% water and 50% methanol	80%	0–10
	73%	10–14		70%	10–13
	68%	14–19		61%	13–18
	60%	19–21		57%	18–20
	48%	21–23		40%	20–22
	40%	23–25		35%	22–24
	35%	1		30%	1
	5%	5		4%	5
	85	4		75	4

**Table 2:** Primers used for qRT-PCR.

Gene	GenBank Accession Number	Primer Sequence (5′-3′)	Product Size (bp)
TNF-α	NM_001278601.1	F: CCCTCACACTCACAAACCAC R: ACAAGGTACAACCCATCGGC	133
IL-6	NM_031168.2	F: ACAAAGCGAGAGTCCTTCAGAG R: GAGCATTGGAAATTGGGGTAGG	108
β-actin	NM_007393.5	F: GTGCTATGTTGCTCTAGACTTCG R: ATGCCACAGGATTCCATACC	174

**Table 3:** Retention time (RT) and peak area (%) of *Rosa damascena* extract’s main constituents.

Peak Name	RT (min)	Area %
Gallic acid	12.113	20.5
Phthalic acid	31.450	21.84
Querctin	32.388	13.12
Apigenin	39.741	11.35
Nonadecane	55.375	30.48
*Β-Sitosterol*	57.056	2.36
*Prasterone acetate*	60.925	0.11
*Eugenol*	61.426	0.23

**Table 4:** Effects of *R. damascena* methanolic extract (100 mg Kg^−1^) on liver enzymes in CdCl_2_ (5 mg.kg^−1^)-treated groups.

Parameters	Control	CdCl_2_	*R. damascena*	CdCl_2_*+* *R. damascena*
ALT (U/L)	13.24 ± 1.26	143.02 ± 5.62 ^a^	13.02 ± 1.01 ^b^	18.02 ± 1.15 ^a,b^
AST (U/L)	12.52 ± 1.46	110.25 ± 7.45 ^a^	12.45 ± 0.34 ^b^	14.17 ± 1.39 ^a,b^

Results are expressed as mean ± SE. ^a^ *p* ≤ 0.001 vs. control group; ^b^ *p* < 0.05 vs. CdCl2 group.

**Table 5:** Effects of *R. damascena* methanolic extract (100 mg Kg^−1^) on oxidative stress enzymes and oxidative damage markers in hepatic tissues in CdCl_2_ (5 mg.kg^−1^) groups.

Parameters	Control	CdCl_2_	*R. damascena*	CdCl_2_*+ R. damascena*
CAT (U/g)	11.02 ± 0.26	2.42 ± 0.16 ^a^	11.05 ± 1.11 ^b^	9.01 ± 0.35 ^a, b^
SOD (U/g)	16.20 ± 0.26	10.07 ± 0.45 ^a^	16.02 ± 0.34 ^b^	14.60 ± 0.39 ^a, b^
GSH (U/g)	15.78 ± 2.38	8.10 ± 0.88 ^a^	16.12 ± 0.33 ^b^	15.64 ± 0.45 ^a, b^
MDA(µg/mg)	3.01 ± 0.15	14.01 ± 1.30 ^a^	2.05 ± 0.68 ^b^	6.01 ± 0.66 ^a, b^

Results are expressed as mean ± SE. ^a^ *p* ≤ 0.001 vs. control group; ^b^ *p* < 0.05 vs. CdCl_2_ group.

**Table 6:** Effects of *R. damascena* methanolic extract (100 mg Kg^−1^) on oxidative stress enzymes and oxidative damage markers in cardiac tissues in CdCl_2_ (5 mg.kg^−1^) groups.

Parameters	Control	CdCl_2_	*R. damascena*	CdCl_2_*+* *R. damascena*
CAT (U/g)	11.20 ± 0.26	3.79 ± 0.16 ^a^	11.02 ± 1.11 ^b^	10.05 ± 0.35 ^a,^ ^b^
SOD (U/g)	13.19 ± 1.26	7.04 ± 1.45 ^a^	13.45 ± 1.34 ^b^	12.87 ± 2.39 ^a,b^
GSH (U/g)	13.10 ± 2.38	7.10 ± 0.88 ^a^	13.22 ± 0.33 ^b^	11.64 ± 0.45 ^a,b^
MDA(µg/mg)	4.87 ± 0.15	18.30 ± 1.30 ^a^	4.25 ± 0.68 ^b^	6.21 ± 0.68 ^a,^ ^b^

Results are expressed as mean ± SE. ^a^ *p* ≤ 0.001 vs. control group; ^b^ *p* < 0.05 vs. CdCl_2_ group.

**Table 7:** *R. damascena leaves extract dose* (100 mg Kg^−1^) ameliorated serum TNF-α and IL-6 in CdCl_2_ (5 mg.kg^−1^) groups. Data are expressed as mean ± SE.

Parameters	Control	CdCl_2_	*R. damascena*	CdCl_2_+ *R. damascena*
TNF-α (pg/mL)	5.69 ± 0.54	22.36 ± 0.21 ^a^	4.36 ± 0.74 ^b^	7.02 ± 1.20 ^a,b^
IL-6 (pg/mL)	3.65 ± 0.14	15.69 ± 1.02 ^a^	3.02 ± 0.69 ^b^	5.25 ± 0.36 ^a,b^

Results are expressed as mean ± SE. ^a^ *p* ≤ 0.001 vs. control group; ^b^ *p* < 0.05 vs. CdCl_2_ group.

## Data Availability

Data generated or analysed during this study are included in this manuscript.
